# Establishment of a high-content compatible platform to assess effects of monocyte-derived factors on neural stem cell proliferation and differentiation

**DOI:** 10.1038/s41598-024-57066-2

**Published:** 2024-05-28

**Authors:** Juliana Campo Garcia, Roemel Jeusep Bueno, Maren Salla, Ivette Martorell-Serra, Bibiane Seeger, Nilufar Akbari, Pia Sperber, Harald Stachelscheid, Carmen Infante-Duarte, Friedemann Paul, Sarah C. Starossom

**Affiliations:** 1 Experimental and Clinical Research Center, a Cooperation Between the Max Delbrück Center for Molecular Medicine in the Helmholtz Association, Charité Universitätsmedizin Berlin, Berlin, Germany; 2 Charité - Universitätsmedizin Berlin, Corporate Member of Freie Universität Berlin and Humboldt-Universität zu Berlin, Experimental and Clinical Research Center, Lindenberger Weg 80, 13125 Berlin, Germany; 3 Max Delbrück Center for Molecular Medicine in the Helmholtz Association (MDC), Berlin, Germany; 4 Institute for Medical Immunology, Charité - Universitätsmedizin Berlin, 13353 Berlin, Germany; 5 Charité - Universitätsmedizin Berlin, Corporate member of Freie Universität Berlin and Humboldt-Universität zu Berlin, Institute of Biometry and Clinical Epidemiology, Charitéplatz 1, 10117 Berlin, Germany; 6 Stem Cell Core Facility, Berlin Institute of Health at Charité - Universitätsmedizin Berlin, Augustenburger Platz 1, 13353 Berlin, Germany; 7 Humboldt-Universität zu Berlin, Faculty of Life Sciences, 10099 Berlin, Germany

**Keywords:** Human neural stem cells, Monocytes, Macrophages, Neuroinflammation, High-content, Gliogenesis, Neurogenesis, Neuroimmunology, Regeneration and repair in the nervous system, Stem cells in the nervous system

## Abstract

During neuroinflammation, monocytes that infiltrate the central nervous system (CNS) may contribute to regenerative processes depending on their activation status. However, the extent and mechanisms of monocyte-induced CNS repair in patients with neuroinflammatory diseases remain largely unknown, partly due to the lack of a fully human assay platform that can recapitulate monocyte-neural stem cell interactions within the CNS microenvironment. We therefore developed a human model system to assess the impact of monocytic factors on neural stem cells, establishing a high-content compatible assay for screening monocyte-induced neural stem cell proliferation and differentiation. The model combined monocytes isolated from healthy donors and human embryonic stem cell derived neural stem cells and integrated both cell-intrinsic and -extrinsic properties. We identified CNS-mimicking culture media options that induced a monocytic phenotype resembling CNS infiltrating monocytes, while allowing adequate monocyte survival. Monocyte-induced proliferation, gliogenic fate and neurogenic fate of neural stem cells were affected by the conditions of monocytic priming and basal neural stem cell culture as extrinsic factors as well as the neural stem cell passage number as an intrinsic neural stem cell property. We developed a high-content compatible human in vitro assay for the integrated analysis of monocyte-derived factors on CNS repair.

## Introduction

Neuroinflammation is a key component of many neurologic and psychiatric diseases including multiple sclerosis, Alzheimer's disease, Parkinson’s disease, stroke, and depression^1–5^. It is characterized by the activation of both, central nervous system (CNS) resident and peripheral immune cells, including blood-derived monocytes, which infiltrate the CNS parenchyma where they contribute to both damaging processes and cellular repair^6,7^. While the destructive role of the immune system in the pathogenesis of neuroinflammatory diseases has long been studied^8–15^, the importance of peripheral immune cells in tissue repair has become more evident in the last decade^6,16,17^.

Endogenous cellular repair mechanisms constitute dynamic processes by which damaged cells are replaced by new cells of the same type. In both, oligodendrogenesis and neurogenesis, cellular repair functions via the expansion, differentiation, and maturation of neural stem cells (NSCs), as well as neural- and glial progenitor cells^18–24^. These cells can migrate and integrate at the site of injury and functionally replace damaged or lost subcellular structures such as synapses (synaptic repair) or myelin (remyelination)^25,26^. In the adult mammalian CNS, these cellular dynamics are essential to maintaining the steady state under physiological conditions and become crucial for cellular repair and functional recovery in the context of neuroinflammation^27–31^.

Given their importance, processes of repair within the CNS are tightly regulated, both by cell intrinsic and extrinsic mechanisms, such as genetic or epigenetic states derived by e.g. aging or microenvironmental factors including cytokines and extracellular matrix, respectively. Yet, endogenous CNS repair often fails, leading to uncontrolled proliferation of NSCs^32,33^ or insufficient or dysregulated repair^34^.

Emerging evidence suggests that during neuroinflammation monocytes infiltrate the CNS and become monocyte-derived cells (MoCs). Monocytes and MoCs are members of the mononuclear phagocyte system and carry out tissue- and microenvironment-specific functions^7^. Their activation influences tissue-protective and pathogenic processes including disease outcomes associated with such functions^7,8,35–42^. During demyelination, the phagocytic properties of MoCs are important for clearing myelin debris, which has inhibitory properties for oligodendrogenesis and remyelination^6,43,44^. Factors secreted by invading monocytes and MoCs within the parenchyma directly promote the differentiation of NSCs, thereby mediating neurogenesis and oligodendrogenesis in a paracrine fashion^29,41,44,45^. However, the specific function carried out by MoCs is tightly connected with their activation status.

Activation states of MoCs range from a pro-inflammatory phenotype associated with cytotoxicity, to a potentially regenerative anti-inflammatory phenotype^16,38,39,46^. Specifically, recent work in mice showed that MoCs can be primed towards a pro-inflammatory or anti-inflammatory phenotype while accumulating at distinct CNS barriers in vivo^38^. Priming and polarization of MoCs depends on the local microenvironment, which may either support a pro-inflammatory phenotype, including the blood brain barrier and active multiple sclerosis lesions^47^ within the CNS parenchyma; or an anti-inflammatory phenotype, as seen in blood-cerebrospinal fluid barriers^38^. The latter may be the default phenotype of myeloid cells within the normal CNS parenchyma^48^. Within this context, anti-inflammatory activated myeloid cells seem to mediate neuroprotective and regenerative functions by secreting pro-regenerative factors that may act on NSCs and parenchymal CNS progenitor cells to differentially induce proliferation, gliogenesis, or neurogenesis^29,36,41,42^.

Myeloid cells, including peripheral monocytes have emerged as new targets for the treatment of neuroinflammatory diseases^49,50^. Yet, the mechanisms by which monocytes induce CNS repair in humans are poorly understood. With emerging therapies that target the myeloid compartment in neuroinflammatory diseases, including Bruton’s tyrosine kinase inhibitors, it is essential to monitor treatment-associated changes affecting the CNS repair potential of patient-derived monocytes. We thus sought to establish a simple short-term assay which can be carried out in a high throughput manner and permits the investigation of immune-mediated effects on NSC differentiation.

## Methods

### Isolation of peripheral blood mononuclear cells (PBMCs) from leukocyte enriched buffy coats, isolation of CD14+ monocytes

All experimental procedures were approved by the local ethics committee and conducted in accordance with the Helsinki Declaration. Leukocyte-enriched buffy coats from anonymous healthy donors were obtained from the German Red Cross after informed consent. For the isolation of PBMCs, buffy coat content was diluted with an equal volume of PBS (1×, without Mg^2+^/Ca^2+^) and centrifuged in a density gradient medium, (density of 1007 g/ml) for 20 min at 760×*g* at room temperature. The PBMC ring was carefully removed, washed in wash medium (5% FCS in RPMI 1640 containing 1% Hepes), resuspended in freezing medium (20% DMSO, 20% FCS, RPMI 1640 containing 1% Hepes) at a density of 5 × 10^6^ cells/ml and stored in liquid N2 until further use. Upon experimental use, PBMCs were thawed, resuspended in thawing medium and counted by trypan blue exclusion. Both dead and live cells were counted to measure viability after thawing. Monocytes were then isolated from thawed PBMCs via positive magnetic activated cell sorting (MACS) using CD14 MicroBeads Human (Miltenyi Biotec, Bergisch Gladbach, Germany) according to manufacturer’s instructions with the exception that half the bead concentration was used. Following cell sorting, cells of both fractions were counted via trypan blue exclusion.

### Phenotypic analysis via flow cytometry

For phenotyping analysis, cells were blocked in FC blocking solution (Miltenyi Biotec, Bergisch Gladbach, Germany) for 15 min at 4 °C, followed by washing and resuspension in PBS. Viability staining (LIVE/DEAD Fixable near infrared stain, Thermo Scientific) was conducted according to manufacturer’s protocol, followed by antibody staining (APC Lineage Cocktail (CD3, CD19, CD20, CD56) BioLegend 363601, FITC CD14 BD 555397) in blocking solution for 15 min at room temperature. Cells were washed and measured on a BD Fortessa Flow Cytometer (BD Biosciences).

### Phenotypic analysis via Real-time quantitative PCR (qPCR)

Total RNA was extracted from CD14+ monocytes/MoCs prior (0 h) and after 48 h of culture in standard Monocyte Medium (MM), StemPro Differentiation Medium (SP) and OPC Differentiation Medium (OPC) using the RNeasy Micro Kit (QIAGEN, Venlo, The Netherlands) according to the manufacturer’s protocol. A two-step qPCR protocol was performed using the High-Capacity cDNA Reverse Transcription Kit (Thermo Fisher Scientific, Massachusetts, USA) and the TaqMan Fast Advanced Mastermix kit (Applied Biosystems, Massachusetts, USA) with 5 μl reaction system according to the manufacturer’s protocol by QuantStudio Real-Time PCR Systems (Thermo Fisher Scientific, Massachusetts, USA). Each reaction was performed in triplicates and normalized to B2M gene expression. The CT value of each well was determined using the QuantStudio Real-Time PCR System software. The relative quantification was determined by arbitrary units (2^−ΔCT^). Primers included the following:

**Table:** 

Primers	Assay ID	Amplicon size (bp)	Company
STAT1	Hs01013996_m1	66	Thermo Fisher Scientific
STAT6	Hs00598625_m1	96	Thermo Fisher Scientific
CCL22	Hs00171080_m1	89	Thermo Fisher Scientific
CSF1R	Hs00911250_m1	74	Thermo Fisher Scientific
CXCR3	Hs01847760_s1	164	Thermo Fisher Scientific
CHI3L1	Hs01072228_m1	62	Thermo Fisher Scientific
CHI3L2	Hs00970220_m1	70	Thermo Fisher Scientific
CHID1	Hs00388156_m1	55	Thermo Fisher Scientific
B2M	Hs00187842_m1	64	Thermo Fisher Scientific

### Immune cell survival assay

Cells were plated at 5 × 10^4^ cells per well in various media to assess viability. Imaging plates (Cellvis P96-1.5H-N) were used, and outer wells were filled with PBS to buffer evaporation. Plates were incubated at 37 °C with 5% CO2 in a humidified atmosphere. Monocyte medium (s. below) was run on all plates as reference. NucGreen (Thermo Fischer) and NucBlue (Thermo Fischer) with final concentrations of 1:250 and 1:45 respectively, were added directly into the medium and incubated for 1 h at 37 °C before imaging. 16 fields of view were imaged and analyzed per well, while a total of 4 wells was analyzed per condition.

### Composition of the monocyte and NSC media

*Monocyte Medium*: RPMI 1640 (Gibco), 10% FCS (Gibco), 10 mM Hepes (Gibco), 2 mM Glutamax (Gibco), 1% Antibiotic/Antimycotic (Gibco); *StemPro Differentiation Medium (SP)*: KnockOut DMEM/F-12 (Gibco), 2% StemPro Neural Supplement (Gibco), 2 mM Glutamax (Gibco), 1% Antibiotic/Antimycotic (Gibco); *OPC Differentiation Medium (OPC Diff)*: OPC Spontaneous Differentiation Media Kit (Sigma-Aldrich SCM106); *OPC Expansion Medium:* Human OPC Expansion Culture Media Kit (Sigma-Aldrich SCM107);

*Neural Differentiation Medium (NDM)*: Neurobasal Medium (Gibco), 2% B27 Supplement (Gibco), 2 mM Glutamax (Gibco), 1% Antibiotic/Antimycotic (Gibco); *B27 Medium*: KnockOut DMEM/F-12 (Gibco), 2% B27 Supplement (Gibco), 2 mM Glutamax (Gibco), 1% Antibiotic/Antimycotic (Gibco); *N2 Medium*: KnockOut DMEM/F-12 (Gibco), 1% N2 Supplement (Gibco), 2 mM Glutamax (Gibco), 1% Antibiotic/Antimycotic (Gibco); *KO DMEM/F-12 Medium*: KnockOut DMEM/F-12 (Gibco), 2 mM Glutamax (Gibco); 1% Antibiotic/Antimycotic (Gibco); *RPMI 1640 Medium*: RPMI 1640 (Gibco), 2 nM Glutamax (Gibco), 1% Antibiotic/Antimycotic (Gibco).

### Passaging and proliferation of hESC derived NSCs

NSCs derived from the human pluripotent stem cell line H9 were purchased from Thermo Fischer Scientific. NSCs were stained with the NSC markers NESTIN, SOX1 and SOX2.

NSCs were washed with cold PBS (without Ca^2+^ and Mg^2+^) and dissociated with pre-warmed StemPro Accutasse (Gibco). Cells were washed with passaging medium (KnockOut DMEM/F-12, 2 nM GlutaMAX, 1% Antibiotic/Antimycotic) in a 1:10 ratio, centrifuged at 300×*g* for 5 min and resuspended in passaging medium. Cells were counted via trypan blue exclusion before plating.

For proliferation, 6-well TC-Treated plastic plates were coated with Geltrex (Gibco), diluted in KnockOut DMEM/F12 (Gibco) with a final concentration of 1:120.

NSCs were proliferated in StemPro Serum Free Proliferation Medium (KnockOut DMEM/F-12, 2% StemPro Supplement, 10 ng/ml EGF, 10 ng/ml FGF2, 2 mM GlutaMAX, and 1% Antibiotic/Antimycotic). Medium was changed every other day until cells reached 100% confluency and were subsequently passaged either for further proliferation or differentiation. Cells used for all experiments came from large batches expanded and frozen at passages (P) 3, 23 and 38 to ensure consistency. Cells were always allowed to proliferate for at least two passages before being used for experiments. Plates were incubated at 37 °C with 5% CO2 in a humidified atmosphere.

### Medium conditioning and conditioned-medium transfer

During medium-conditioning, both “Conditioned” and “Non-Conditioned” media were plated side by side on the same plates and incubated together. All the handling was identical for both conditions apart from the presence of CD14+ cells. More specifically, for the “Conditioned” medium CD14+ cells were plated at 2 × 10^5^ cells in 200ul medium per well in 96-well TC-treated plates. Adjacent wells within the same plate were used for the generation of “Non-Conditioned” medium by adding 200ul of medium to empty wells. Plates containing both conditions, ensuring any effects not related to the cells themselves (such as incubation and handling) were controlled, were incubated for 48 h at 37 °C with 5% CO2 in a humidified atmosphere. Supernatants were collected, centrifuged, and stored at − 80 °C prior to conditioned-medium transfer experiments. Again, this was all carried out for both conditions. Cells (only for “Conditioned”) were lysed for qPCR analysis.

For conditioned-medium transfer experiments “Conditioned” and “Non-Conditioned” media were plated in adjacent wells in order to avoid intra-plate variation. Outer wells were never used for experiments but were nonetheless treated the same as the rest of the plate (coated, seeded with cells, and medium changed in accordance with protocol) in order to avoid evaporation issues.

### Differentiation of hESC derived NSCs

For differentiation, 384-well Cell Carrier Ultra Plates (Perkin Elmer) were coated with Poly-d-Lysine (70,000–150,000 MW, Sigma-Aldrich) diluted in H_2_O (0.1 mg/mL) overnight at 4 °C and subsequently washed with H_2_O. Plates were then coated with Laminin (Natural Mouse, Gibco) diluted in H_2_O (0.01 mg/ml) overnight at 4 °C and subsequently washed with PBS.

25ul of medium were added to wells directly after coating onto which cells were then seeded in a further 25ul of medium resulting in a better distribution of cells. Plates were incubated at 37 °C with 5% CO_2_ in a humidified atmosphere. The following day an additional 50ul of medium was added containing the conditioned medium. This was considered day 0 of differentiation. At day 4 of differentiation 80% of medium was changed. At day 8 the differentiation was stopped, and cells were fixed. Due to a logistical issue, one differentiation experiment was not stopped at day 8 but instead at day 9. This change in protocol was included as a random effect in the statistical analysis conducted and summarized in Table 1 (see statistics methods).

**Table 1:** Linear mixed model analyzing associations of experimental culture conditions (predictors) on monocyte-induced NSC proliferation (KI67), gliogenesis (A2B5) and neurogenesis (DCX); reported estimated 95% confidence interval (CI) and p-value.

Predictors	%_KI67			%_A2B5			%_DCX		
	Estimates	CI	*p*	Estimates	CI	*p*	Estimates	CI	*p*
(Intercept)	0.79	− 0.33 to 1.90	0.167	0.20	− 0.56 to 0.97	0.598	− 0.22	− 1.16 to 0.71	0.639
MoC conditioning [Cond]	− 0.20	− 0.48 to 0.08	0.167	0.39	0.24 to 0.54	** < 0.001**	0.07	− 0.12 to 0.25	0.494
Conditioned medium [Mono]	− 1.30	− 1.58 to − 1.01	** < 0.001**	− 1.68	− 1.83 to − 1.53	** < 0.001**	− 1.06	− 1.25 to − 0.88	** < 0.001**
Conditioned medium [OPC]	− 0.41	− 0.69 to − 0.13	**0.005**	0.19	0.03 to 0.34	**0.018**	0.45	0.26 to 0.63	** < 0.001**
Conditioned medium [SP]	− 0.54	− 0.82 to − 0.26	** < 0.001**	− 0.02	− 0.17 to 0.13	0.803	0.30	0.11 to 0.49	**0.002**
Basal medium [N2]	3.08	2.90 to 3.27	** < 0.001**	1.11	1.02 to 1.20	** < 0.001**	1.42	1.31 to 1.54	** < 0.001**
Basal medium [NDM]	0.11	− 0.07 to 0.30	0.239	− 0.05	− 0.15 to 0.04	0.274	− 0.08	− 0.19 to 0.04	0.188
Percentage	− 0.01	− 0.01 to 0.00	0.234	− 0.00	− 0.01 to 0.00	0.063	0.00	− 0.00 to 0.01	0.510
MoC conditioning [Cond] * conditioned medium [Mono]	0.12	− 0.28 to 0.52	0.547	− 0.27	− 0.48 to − 0.05	**0.015**	0.14	− 0.13 to 0.41	0.308
MoC conditioning [Cond] * conditioned medium [OPC]	− 0.94	− 1.34 to − 0.54	** < 0.001**	0.22	0.01 to 0.44	**0.042**	1.15	0.88 to 1.42	** < 0.001**
MoC conditioning [Cond] * conditioned medium [SP]	− 0.83	− 1.23 to − 0.43	** < 0.001**	0.33	0.12 to 0.55	**0.003**	0.40	0.13 to 0.66	**0.004**
Random effects									
σ^2^	3.07			1.15			1.75		
τ_00_	0.92 _Experiment_			0.44 _Experiment_			0.66 _Experiment_		
	0.00 _Diff_Days_			0.00 _Diff_Days_			0.00 _Diff_Days_		
N	3 _Experiment_			3 _Experiment_			3 _Experiment_		
	2 _Diff_Days_			2 _Diff_Days_			2 _Diff_Days_		
Observations	2352			3024			3024		
Marginal R^2^/Conditional R^2^	0.423/NA			0.487/NA			0.394/NA		

Significant values are in bold.

### Fixation and staining protocols

80 μl of medium was removed from each well and 20 μl of pre-warmed (37 °C), 8% PFA was added, resulting in a total volume of 40 μl and final concentration of 4% PFA. Plates were incubated for 15 min at 37 °C, then washed twice with PBS before proceeding with staining.

To establish the panel of markers to characterize the NSCs, a comprehensive list of antibodies was tested, covering the three lineages in various stages of differentiation. From these, a final panel was chosen based on the expression of the markers, the robustness of the quantification and their compatibility with each other. For immunohistochemical staining, cells were blocked for one hour at room temperature with 5% BSA and 5% Horse serum in PBS (without Ca^2+^ and Mg^2+^). Primary antibodies including A2B5 (R&D Systems, MAB1416), DCX (Novus Biological, NBP1-72042), KI67 (Abcam, Ab16667), NG2 (BD 554275), OLIG2 (Millipore, AB9610), SOX10 (R&D Systems, AF2864), GALC (Merck, MAB342), PDGFRɑ (CST, 5241T), O4 (R&D Systems, MAB1326), NEUN (CST, 24307), PLP (Sigma Aldrich, MAB388), MBP (Sigma Aldrich, AB9348), βIIITUB (Biolegend, 801213), GFAP (Agilent, Z0334), CASPASE3 (R&D Systems, MAB835), PCNA (Santa Cruz Biotechnology, sc-56), NESTIN (R&D Systems, MAB1259), SOX1 (R&D Systems, AF3369) and SOX2 (R&D Systems, AF2018) were incubated in blocking solution at 4 °C overnight. Secondary antibodies were incubated in diluted blocking solution (2.5% BSA and 2.5% horse serum) containing DAPI (50 ng/mL) and incubated for one hour at room temperature. Cells were washed and imaged in PBS.

### Microscopy

Imaging of fluorescent staining was done with the High Content Screening System Opera Phenix (Perkin Elmer), with the 20× water objective, in confocal mode. For the immune cell survival assay live imaging settings with 37 °C and 5% CO2 were used. All image analysis was done with the Columbus Image Analysis server (Perkin Elmer). Imaging of neurosphere formation assays was done with a Leica DMI6000B microscope.

### Columbus image analysis

For the quantification of immune cell viability after 48 h in culture with different media the analysis pipeline was constructed as follows. For the input image, the available flatfield correction was applied. First nuclei were identified in the NucBlue channel, using Method C. Next, morphology (Method : Standard, Area, Roundness) and intensity of NucGreen (Method : Standard, Mean) were calculated. Then a surrounding region around each nucleus was found using method A, and the intensity of the NucGreen channel was also calculated for this region (Method : Standard, Mean). Next a ratio was calculated between the mean intensity of each nucleus and the mean intensity of the surrounding region (Method : By Formula, Formula : A/B, Variable A : Intensity Nucleus Alexa 488 Mean, Variable B : Intensity Surrounding Region Nucleus Alexa 488 Mean). This was done in order to correct for the differences in background fluorescence due to the composition of the different media utilized. Then nuclei were selected (Method : Filter by Property, Nucleus Area, Nucleus Roundness) excluding artifacts, and lastly dead nuclei were identified (Method : Filter by Property, Nuclei Selected, Ratio Nucleus/Surrounding Region).

For the quantification of antibody stainings (KI67, A2B5, DCX, NG2) the analysis pipeline was constructed as follows. For the input image, the available flatfield correction was applied. First nuclei were identified in the DAPI channel, using Method B. Next, morphology (Method : Standard, Area, Roundness) and intensity of DAPI (Method : Standard, Mean) were calculated. At this point viable nuclei were selected (Method: Filter by property Nucleus Area, Nucleus Roundness, Intensity Nucleus DAPI) in order to exclude dead nuclei and artifacts, and all following steps were done with this nucleus population (live nuclei). Intensities of nuclear stainings (KI67, DCX) were calculated (Method: Standard, Mean). While DCX is not per se a nuclear staining, the signal within the nuclear region was always very bright and reliable, which is why we opted to quantify it in this manner. For cytoplasmic stainings (A2B5, NG2) the cytoplasm was identified using Method B, and the respective intensities (Method: Standard, Mean) and morphology (Method : Standard, Area, Roundness) calculated. Cell Area and Roundness were used to not only take staining intensity but also cellular morphology into account in order to refine the analysis and further avoid potential artifacts. Lastly, positive cells were identified (Method : Filter by Property) using the appropriate properties for each antibody of interest (Such as Nucleus Intensity, Cytoplasm Intensity, Cell Area and Cell Roundness).

### Neurosphere formation assay

NSCs were plated in non-coated tissue culture plates (6well) at a density of 1 × 10^5^ cells/well. Cells were plated in 2 ml proliferation medium and 1 ml of fresh proliferation Medium was added every two days. Neurospheres were allowed to form until day 5–7, at which time 10 random images were taken throughout each well. The number of neurospheres per image as well as the individual diameter of each neurosphere was then quantified with ImageJ (Rasband, W.S., ImageJ, U. S. National Institutes of Health, Bethesda, Maryland, USA, https://imagej.nih.gov/ij/, 1997–2018).

### Statistics

Graphs and statistical analyses were done using GraphPad Prism version 9.0.0 for Windows, GraphPad Software, San Diego, California USA, www.graphpad.com, or R version 4.0.2 (2020-06-22) (R Core Team (2021). R: A language and environment for statistical computing. R Foundation for Statistical Computing, Vienna, Austria. URL https://www.R-project.org/).

**Figure 1:**
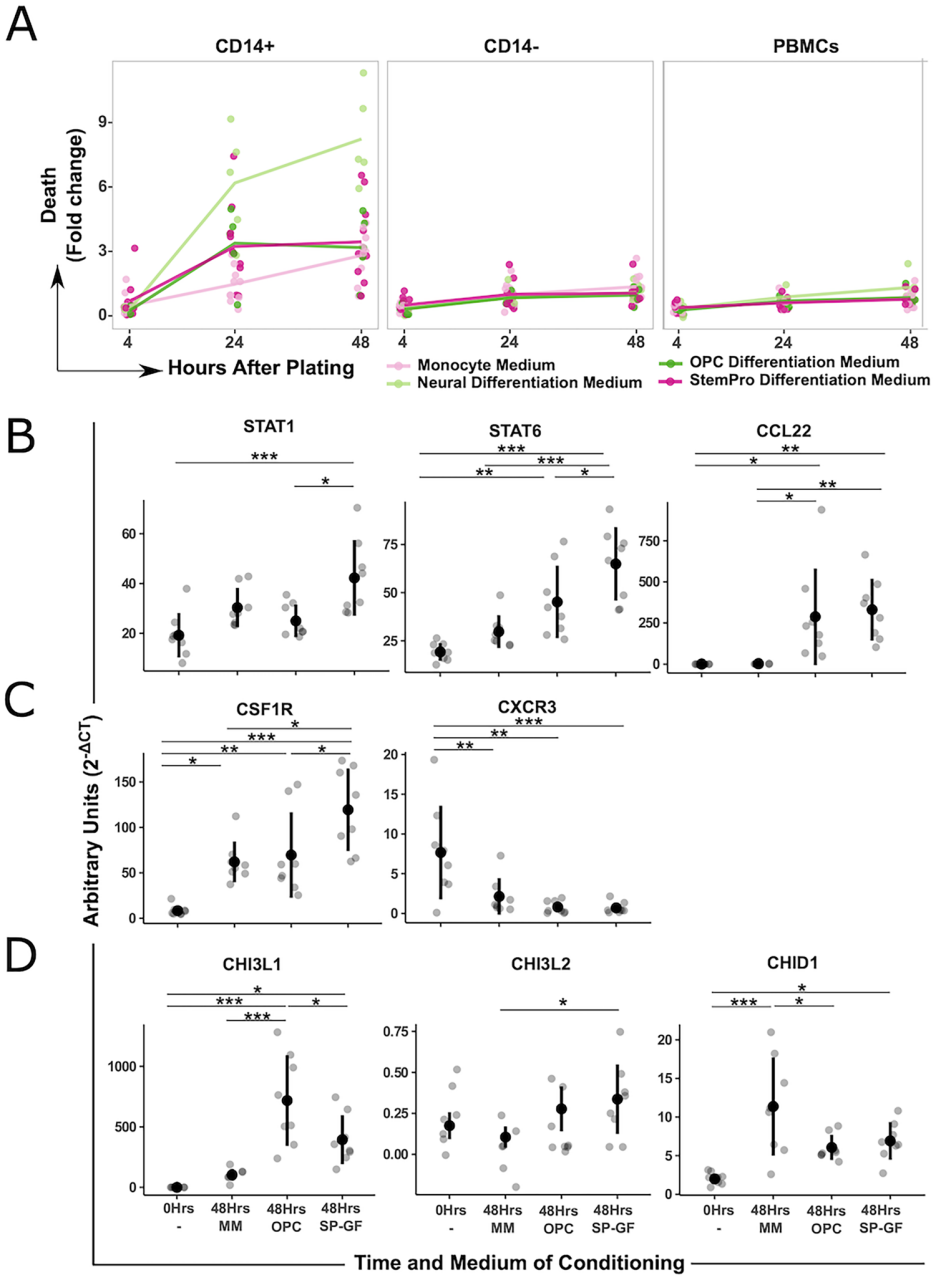
Survival and activation of immune cells in various media. (**A**) Cell death of CD14+, CD14− cells and PBMCs in different media over time. Each dot represents the average of four wells (96 well plate) for each healthy donor (n = 10 healthy donors). Data was analyzed by comparing all media to the reference MM, Dunnets’s 95% CI can be found summarized in Supplementary Fig. 2. (**B**)–(**D**) Gene expression of (**B**) M1/M2 polarization markers, (**C**) maturation, homing and CNS infiltration markers, and (**D**) Chitinase-like proteins, measured by qPCR in CD14+ cells directly after isolation (0 h) and after 48 h in different media. Each dot represents one healthy donor (n = 8). Kruskal Wallis one way ANOVA, followed by Tukey’s HSD post-hoc test. *p < 0.05; **p < 0.01; ***p < 0.001.

The evaluation must be understood as explorative, therefore p-values do not allow confirmatory conclusions. Due to the small sample size in the gene expression data of MoCs in culture with different media (Fig. 1B–D) and staining of cells in different passages (Fig. 3C) non-parametric Kruskal Wallis one way ANOVA was used for continuous endpoints followed by Tukey’s honestly significant difference (HSD) test.

Immune cell viability and purity following MACS as well as neurosphere number and diameter were summarized descriptively. For these continuous variables, descriptive statistics included mean and standard deviation (SD) and data were displayed in Supplementary Fig. 1 and Fig. 3.

**Figure 2:**
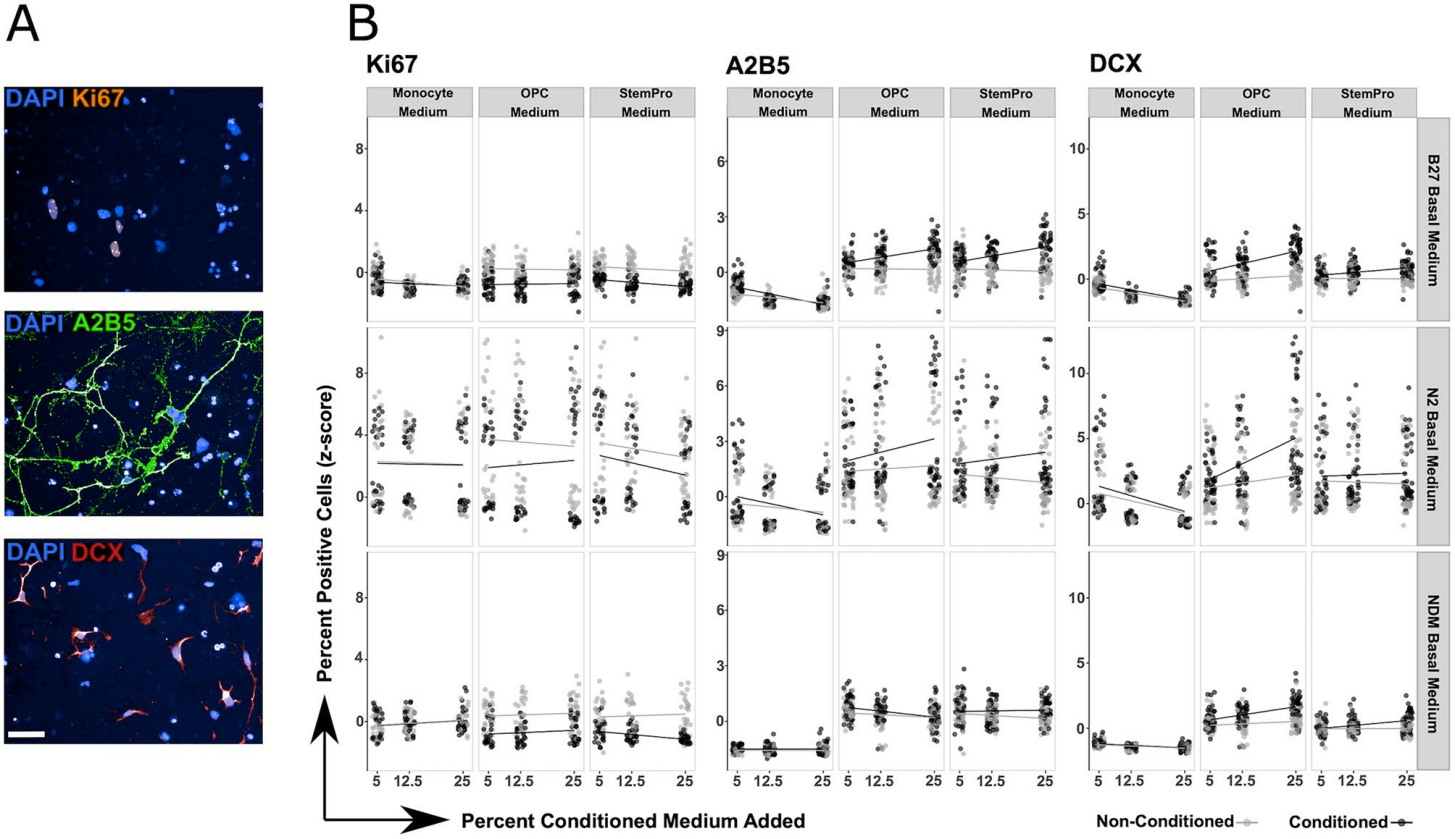
MoC-induced NSC proliferation and differentiation is dependent on monocytic and neural stem cell microenvironment. (**A**) Representative images of KI67 (Orange), A2B5 (Green) and DCX (Red) after differentiation. White bar is 50 µm long. (**B**) NSC differentiation as measured by the differentiation markers A2B5 and DCX as well as the proliferation marker KI67. Three different conditioned media (listed at the top) were tested in combination with three different basal media (listed on the right). Each conditioned medium was added at 5%, 12.5% or 25% (percentage shown on x axis). Each dot represents the average number of positive cells in one well (384 well plate). Three independent experiments were performed, each with 16 wells per condition. Results were normalized (z-score normalization) and analyzed together, using mixed linear models (results summarized in Table 2).

The data depicted in Fig. 2, shows three independent experiments that were standardized with a z-score normalization to make the changes comparable.

Linear mixed models (LMM) were used to analyze the association between experimental culture conditions and monocyte-induced NSC proliferation (KI67), gliogenesis (A2B5) and neurogenesis (DCX). LMM analysis was done with the “lme4” R package^51^. For the data presented in Fig. 2 models were used described by the equation: % Positive Cells ~ MoC Conditioning + Conditioned Medium + Basal Medium + Conditioning * Conditioned Medium + Percentage + (1| Experiment) + (1|Differentiation Days). Fixed effects included MoC Conditioning, Conditioned Medium, Basal Medium and Percentage. Random effects included Experiment and differentiation days. For the data presented in Fig. 4 models were used described by the equation: % Positive Cells ~ MoC Conditioning + Conditioned Medium + NSC Passage + MoC Condition * NSC Passage + (1| Healthy Donor) . Fixed effects included MoC Conditioning, Conditioned Medium and NSC Passage. Random effect was defined as Healthy Donors.

### Ethics approval and consent to participate

All experimental procedures were approved by the local ethics committee and conducted in accordance with the Helsinki Declaration. Leukocyte-enriched buffy coats from anonymous healthy donors were obtained from the German Red Cross after informed consent.

## Results

### Activation of human monocytes/MoCs in vitro: Survival and characterization in response to culture conditions mimicking the bloodstream vs. the CNS parenchyma

To develop an optimized in vitro assay that models MoC-mediated CNS repair in a dish, we integrated the microenvironmental component of CNS parenchyma, in which MoCs interact with neural progenitor cells in a paracrine fashion. We defined optimal culture conditions as ones that are serum free and optimized for the growth and differentiation of CNS progenitor cells, without compromising the survival and activation capacity of monocytes. This allowed the analysis of myeloid-mediated effects on NSC proliferation and differentiation with high reproducibility in a high throughput format.

Protocols were established using CD14+ monocytes that were isolated from cryopreserved PBMCs, which were collected from 10 healthy donors with the following demographics: 5 × female, mean age of 48.8 (SD 5.17); 5 × male, mean age of 51.2 (SD 14.1). The mean viability of PBMCs after thawing, measured via trypan blue exclusion was ≥ 95.00%.

CD14+ monocytes were isolated using magnetic beads for positive selection, ensuring high viability (> 97.95%) and high purity of the enriched CD14+ cell population (> 92.58% lineage negative cells, > 97.27% CD14+ cells) (Supplementary Fig. 1). For this section, we will refer to whole PBMCs prior MACS sorting as “PBMCs” and the positive and negative fractions of CD14+ MACS sorting, as “CD14+” and “CD14−” cells respectively, MoCs refers to CD14+ cells in culture.

Our first aim was to determine the optimal CNS-mimicking culture conditions, by utilizing media that are normally used for neural cell culture, (see methods section) in which CD14+ cells retained adequate survival rates for up to 48 h, when compared to the standard Monocyte Medium (MM), which mimics the microenvironment of the blood stream.

CD14+ cells cultured in StemPro Differentiation Medium (SP) and OPC Differentiation Medium (OPC) for 48 h showed adequate cell survival rates with low variance across experiments, while Neural Differentiation Medium (NDM) showed reduced survival after 24 and 48 h of culture compared to MM (Fig. 1A, left panel). Interestingly, these differences in overall survival and variance were not seen in the CD14− cells (Fig. 1A, middle panel) or PBMCs (Fig. 1 A right panel), indicating a specific vulnerability of the CD14+ monocyte population to microenvironmental changes. Other media that showed adequate overall survival rates but higher variability across all three cell populations (PBMCs, CD14+ and CD14 −) included N2 Medium, OPC Proliferation Medium, KO DMEM/F-12 Medium, and RPMI 1640 Medium (Dunnett’s 95% confidence intervals comparing each medium tested to the reference MM can be found summarized in Supplementary Fig. 2). Based on adequate cell survival with low variance across donors, our results identified SP and OPC Media as optimal for the conditioning of primary human CD14+ monocytes in CNS-mimicking microenvironmental conditions.

### CD14+ cell profiling after 48 h in different media

Next, we examined the activation state of CD14+ monocytes/MoCs prior (0 h) and after 48 h of culture in MM, SP, OPC media based on gene expression of seminal activation and polarization markers. STAT1 was chosen as a marker for a proinflammatory/M1 activation state and STAT6 and CCL22 as markers of an anti-inflammatory/M2 activation.

MoCs cultured in MM for 48 h compared to CD14+ cells at 0 h did not result in significant up regulation of M1 or M2 activation markers (Fig. 1B), SP and OPC medium showed an upregulation of both M2 markers, STAT6 and CCL22 relative to MoCs cultured in MM, indicating that these two CNS-mimicking culture conditions drive priming of MoCs towards the M2 phenotype (Fig. 1B). Additionally, SP medium led to an upregulation of STAT1 expression relative to both 0 h and OPC medium, indicating that SP culture conditions allowed either for an M1/M2 intermediate/mixed activation phenotype, or a heterogeneous M1/M2 activation phenotype among distinct subpopulations of cultured MoCs (Fig. 1B).

Further, we looked at markers of maturation, homing and infiltration of the CNS parenchyma CSF1R and CXCR3. CSF1R gene expression showed an increase in MM, OPC and SP media compared to 0 h. MoCs cultured in SP also upregulated CSF1R gene expression compared to MoCs cultured in MM (Fig. 1C). On the contrary, CXCR3 was decreased in MM, OPC and SP media compared to CD14+ monocytes at 0 h.

Lastly, we looked at the expression of the human chitinase like proteins CHI3L1, CHI3L2 and CHID1. Chitinase like proteins are expressed by immune cells including monocytes, macrophages and microglia^52^ and have been linked to neuroinflammatory processes including tissue remodeling^29^. Here, we saw an increase in CHI3L1 expression in both OPC and SP differentiation media compared to 0 h and a significant increase in OPC media compared to MM. Furthermore, we saw a modest increase in CHI3L2 expression induced by SP differentiation medium compared to MM (Fig. 1D). Additionally, a trend of higher expression of CHI3L2 in OPC and SP media compared to MM was seen (Fig. 1D).

Lastly, MM resulted in an increase in the expression of CHID1 compared to 0 h (Fig. 1D).

Taken together, we conclude that both neuronal cell culture media, OPC and SP induce a phenotype resembling monocytes infiltrating the brain parenchyma without compromising cell viability after 48 h in culture, while MM, a standard medium for the culture of monocytes induces or rather retains a phenotype that resembles that of circulating monocytes. Thus, to investigate the role of monocyte/MoCs on NSC differentiation and proliferation, OPC and SP media will better mimic the physiological conditions.

### MoC conditioned media exert different effects depending on basal medium

The microenvironment not only primes myeloid cells to secrete modulating factors, but it also directly acts on NSCs to influence their susceptibility for modulation of differentiation capacity and self-renewal by exogenous factors^29,41,53,54^.

Here, we systematically tested multiple microenvironmental culture parameters for their ability to demonstrate MoC-mediated NSC-differentiation capacity and self-renewal, while keeping a low variance among technical replicates.

Culture parameters tested included (1) the type of differentiation media that was used to generate MoC-conditioned media (SP, OPC, MM); (2) the type of NSC differentiation media (basal media) to which conditioned media was added for the culture of NSCs (B27, N2 and NDM) and (3) the percentage at which MoC-conditioned media was added to NSC basal media (5%, 12.5%, 25%) for the culture of NSCs. Additionally, MoC-conditioned media was compared to control media, which was not conditioned by MoCs (non-conditioned) but treated equally during conditioning. After differentiation, NSCs were stained with the differentiation markers A2B5 and DCX, markers for glial and neuronal precursors respectively, as well as the proliferation marker KI67. The data were analyzed with a mixed linear model, which allowed us to take into account variability between as well as within experiments (see methods) (Table 1).

MoC-conditioned SP and OPC differentiation media induced a dose-independent decrease in proliferation in all three basal media (Fig. 2 columns 2&3), while the inability of conditioned MM to alter the proliferation capacity of NSCs was independent of the chosen NSC basal medium.

The percentage of NSCs that differentiated towards A2B5 + glial precursor cells increased in response to conditioned SP and OPC differentiation media in a dose dependent manner, however this effect was only observable in B27 and N2 basal media (Fig. 2 columns 5&6). Furthermore, the percentage of NSCs that differentiated towards DCX + neural precursor cells increased in a dose dependent manner (with increasing percentage of MoC-conditioned medium added) in conditioned SP and OPC differentiation media in combination with B27 and N2 basal media (Fig. 2 columns 8&9). Importantly, both conditioned and non-conditioned MM, led to a dose dependent decrease in neuro- and gliogenesis in B27 and N2 basal media, but showed no effect in NDM.

Notably, NSCs differentiated in N2 basal medium showed a vast variance among technical replicates in all staining markers and across all culture conditions (Fig. 2 middle row), and thus N2 differentiation medium was ruled out as NSC basal medium due to its incompatibility with high-content analysis requirements. NDM basal medium only allowed modulation of proliferation but not NSC differentiation, while B27 basal medium allowed MoC-mediated effects on NSCs, both on proliferation and differentiation.

Taken together, these results show that factors secreted by MoCs can induce a decrease in proliferation accompanied by the differentiation of NSCs, which depends on the type of medium used for monocyte conditioning and NSC differentiation. Importantly the effects of MM on NSC differentiation and proliferation were the same in both conditioned and non-conditioned medium, unlike SP and OPC media, indicating that MM itself affects NSC proliferation and differentiation and should not be used in conditioned medium transfer assays with NSCs.

### Passage number influences NSC differentiation potential without affecting stemness

It has been previously reported that NSCs of higher passage number have a greater tendency in fate choice towards the glial lineage compared to NSCs of lower passages^55^. This allows for the rapid generation of glial progenitors in shorter time spans and the establishment of models optimized for the investigation of glial biology.

**Figure 3:**
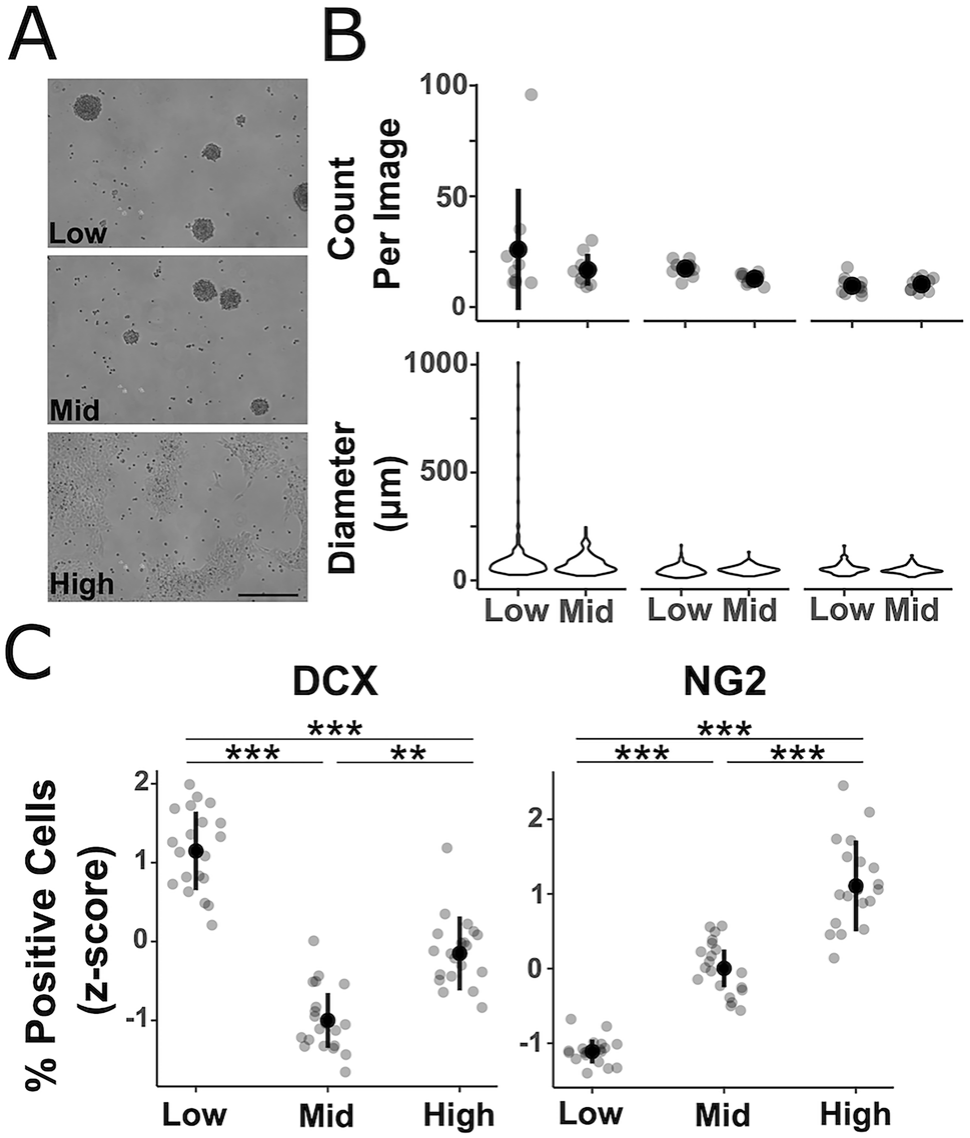
Passage-dependent intrinsic properties affect self-renewal and differentiation capacity of NSCs. (**A**) Representative images of a neurosphere formation assay with low, middle and high passage cells. High passage cells do not form neurospheres. (**B**) Quantification of neurosphere formation assays. Neurospheres from low passage cells do not differ from neurospheres formed by middle passage cells in their quantity or size (top and bottom panels respectively). Data described by summary statistics including mean and SD for neurosphere count and violin plot for neurosphere diameter (top and bottom panels respectively). Each dot represents one image. Neurosphere formation assay was performed three times, each time ten random images were taken (n = 3). (**C**) Quantification of differentiation markers DCX and NG2 after differentiation with low, middle and high passage cells. With increasing passage number cells differentiate more likely into the glial lineage while decreasing their differentiation into the neuronal lineage. Each dot represents one well (96 well plates); two independent experiments with 10 wells per passage were performed, standardized (z-score standardization) and plotted together. Statistics were performed for each individual experiment and largest p-values are displayed. Kruskal Wallis one way ANOVA, followed by Tukey’s HSD post-hoc test. *p < 0.05; **p < 0.01; ***p < 0.001.

Thus, we next tested the effect of passage number of NSCs on stemness (self-renewal capacity) and differentiation capacity. Stemness of NSCs was tested by immunocytochemical means as well as their neurosphere forming capacity. Low passage cells were defined as cells below passage (P) 10, middle passage cells were those between P20 and P30, and high passage cells were those above P40. While we saw no difference between low and middle passage NSCs in their ability to form neurospheres (neither in number nor size), high passage cells grew fully adherent (Fig. 3A,B), indicating that only the stemness of low and middle passage cells can be assured and higher passage cells should not be considered for this assay. Interestingly, no difference in the expression of NSC markers NESTIN, SOX1 and SOX2 was observed between any of the passages (data not shown).

In accordance with the data of Alisch et al.^55^ differentiation of NSCs with increasing passage number resulted in higher percentages of NG2 positive cells (Fig. 3C). Contrarily, we observed the highest percentage of DCX + neuroblasts in low passage followed by high and middle passage NSCs. Taken together, NSCs retain their stem cell character until middle passage, while low and middle passage cells may be distinctly utilized in assaying neurogenesis and gliogenesis in vitro, respectively.

### Effects of MoC-conditioning is dependent on passage number

Next, we investigated the effects of NSC-intrinsic parameters (passage number) on NSC proliferation and differentiation in response to MoC-conditioned supernatants using the above-described assay. Specifically, we analyzed proliferation (KI67), gliogenesis (A2B5) and neurogenesis (DCX) of low and middle passage NSCs, cultured in 25% OPC or SP conditioned media in B27 basal medium for 8 days. Data was analyzed with a mixed linear model and the results are summarized in Table 2.

**Table 2:** Linear mixed model analyzing associations of experimental culture conditions (predictors) and NSC passage number on monocyte-induced NSC proliferation (KI67), gliogenesis (A2B5) and neurogenesis (DCX); reported estimated 95% confidence interval (CI) and p-value.

Predictors	%_KI67			%_A2B5			%_DCX		
	Estimates	CI	*p*	Estimates	CI	*p*	Estimates	CI	*p*
(Intercept)	4.67	4.00 to 5.34	** < 0.001**	5.75	4.94 to 6.55	** < 0.001**	25.55	21.81 to 29.29	** < 0.001**
MoC conditioning [Cond]	− 2.05	− 2.84 to − 1.25	** < 0.001**	2.15	1.31 to 2.98	** < 0.001**	17.87	14.68 to 21.06	** < 0.001**
Conditioned medium [SP-GF]	− 0.25	− 0.82 to 0.31	0.372	0.40	− 0.19 to 0.98	0.184	0.06	− 2.18 to 2.31	0.955
NSC passage [Mid]	1.23	0.44 to 2.03	**0.003**	− 2.36	− 3.19 to − 1.53	** < 0.001**	− 6.61	− 9.77 to − 3.45	** < 0.001**
MoC conditioning [Cond] *passage [Mid]	0.20	− 0.92 to 1.33	0.720	− 3.36	− 4.53 to − 2.18	** < 0.001**	− 6.55	− 11.04 to − 2.05	**0.005**
Random effects									
σ^2^	2.42			2.61			38.25		
τ_00_	0.07 _BC_			0.28 _BC_			9.84 _BC_		
ICC	0.03			0.10			0.20		
N	5 _BC_			5 _BC_			5 _BC_		
Observations	120			119			119		
Marginal R^2^/conditional R^2^	0.364/0.381			0.627/0.663			0.625/0.702		

Significant values are in bold.

**Figure 4:**
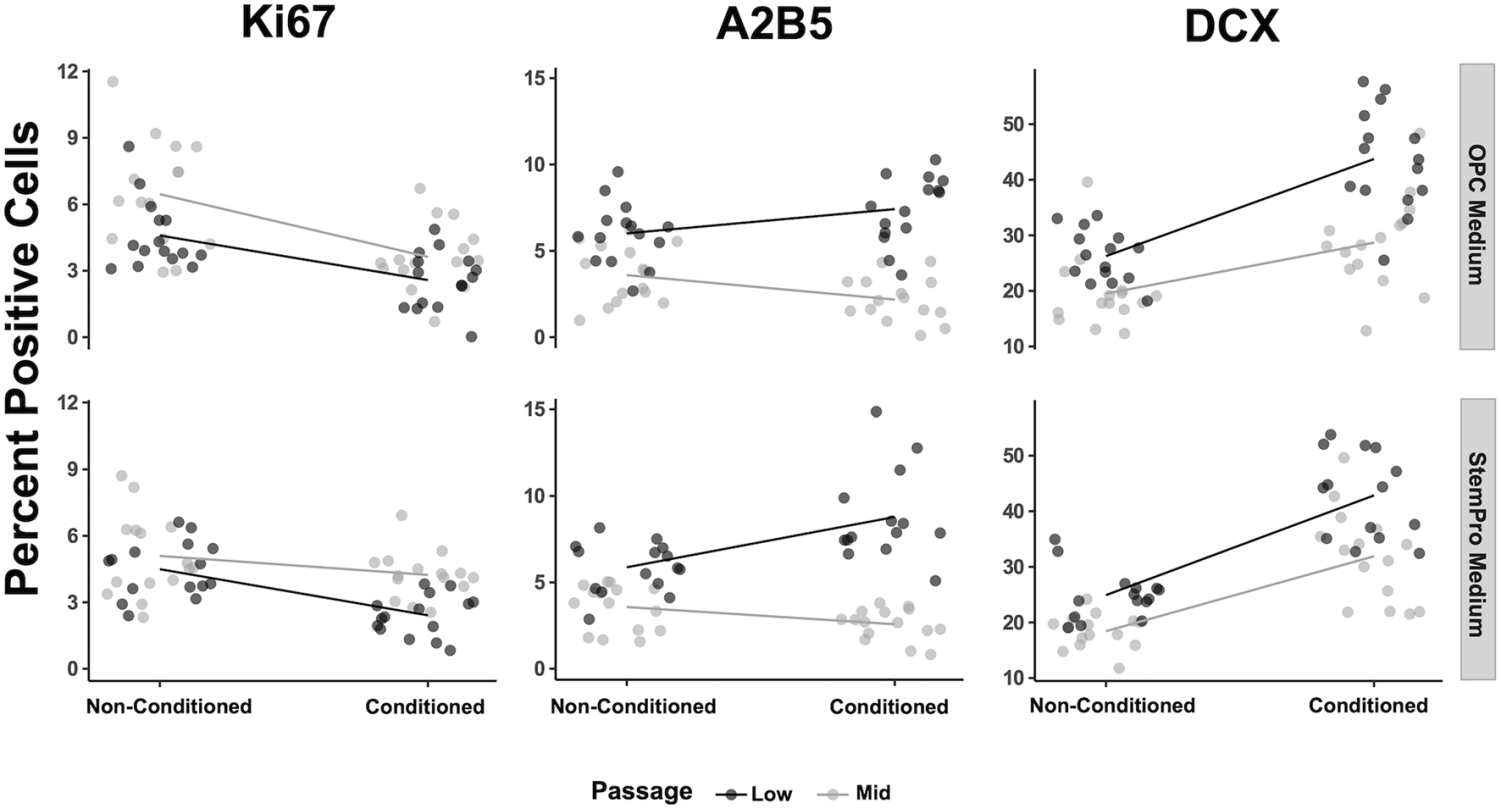
NSC-intrinsic properties influence susceptibility to monocyte/MoC-mediated NSC proliferation and differentiation. Quantification of KI67, A2B5 and DCX after differentiation in monocyte conditioned media. OPC (top row) and SP (bottom row) conditioned media were tested in low (black) and middle (gray) passage NSCs. Conditioned medium was added at 25%. Each dot represents the average number of positive cells in one well (384 well plate). Experiment was performed including n = 5 healthy donors, each with 3 wells (technical replicates) per condition. Results were normalized (z-score normalization) and analyzed together, using mixed linear models (results summarized in Table 2).

Consistent with our previous findings, we observed that conditioned medium induced a decrease in proliferation and increase in neurogenic (DCX) differentiation in both low and medium passage NSCs (Fig. 4). The effect on glial differentiation (A2B5), however, was only observed in low passage cells, but not medium passage cells, indicating that in addition to the niche microenvironment, NSC-intrinsic factors influence their susceptibility to monocyte/MoC-induced glial differentiation also in vitro.

## Discussion

**Figure 5:**
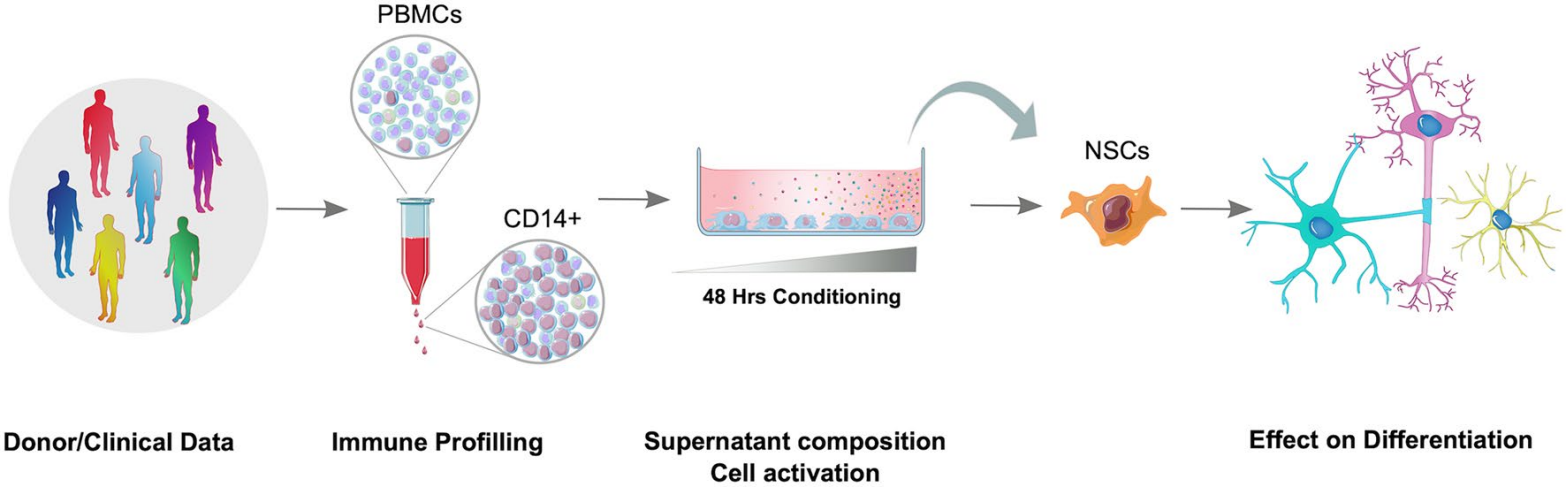
Illustration of proposed assay for studying the effects of CNS-infiltrating monocytes on NSC differentiation and proliferation in humans.

In this study we developed an assay for the integrated analysis of human MoC-mediated CNS repair in vitro, paying particular attention to parameters likely to be taken for granted when setting up stem cell-related in vitro assays such as type of medium or cell passage number (Fig. 5). Our proposed assay has the capacity to closely mimic the microenvironment of the germinal niches and sites of regeneration in the CNS in the context of neuroinflammation.

The adult germinal niches in the CNS include the subventricular zone and hippocampal dentate gyrus. These intricate niches harbor endogenous NSCs that have the capacity to contribute to cellular repair through proliferation and differentiation towards neurons, astrocytes, and oligodendrocytes^56^. Such cellular repair depends on factors intrinsic to the NSC compartment such as genetic or epigenetic alterations due to repeated cell turn over/aging, and extrinsic factors, including the niche microenvironment^54^.

Within the context of neuroinflammation monocytes infiltrate the CNS, and their progeny contributes to both degenerative as well as regenerative processes, depending on their activation phenotype^7,8,37,54^. Thereby, monocytes and MoCs create the microenvironment on which the efficacy and direction of stem cell-mediated repair depends.

Importantly, monocytes infiltrate the CNS via distinct barriers where they functionally polarize into either neurotoxic pro-inflammatory MoCs or regenerative anti-inflammatory MoCs^7,38,46^. Modeling human MoC-induced CNS repair in a CNS-mimicking microenvironment may thus aid to reveal immune-centered mechanisms for the development of CNS-regenerative therapies.

In this study, we use human primary monocytes and human embryonic stem cell derived NSCs to model human MoC-induced CNS repair. Here, we identify two cell culture media, SP and OPC differentiation media that induce a MoC-phenotype closely resembling the activation state expected of CNS-infiltrating monocytes while allowing adequate monocyte survival in culture. Compared to the standard MM, CD14+ monocytes cultured in SP medium acquired a tissue-infiltrating anti-inflammatory phenotype, which was marked by increased expression of M2 signature genes STAT6, CCL22 and CSF1R, as a marker of macrophage maturation and CNS tissue infiltration. Notably, CSF1R signaling is required for survival, proliferation and differentiation of myeloid cells and its expression is gradually increased along the differentiation stages of monocytes towards tissue macrophages^57^, with a trophic anti-inflammatory (M2) like phenotype^58^.

Within the myeloid lineage, an increase in CSF1R expression is associated with myeloid cell differentiation and chemotaxis towards tissue-derived ligands^57,59^. Contrarily, CXCR3 is highly expressed in peripheral monocytes and is involved in monocyte transmigration into the CNS, where enhanced receptor-ligand binding leads to internalization of the receptor^60–64^. In line with these findings, we observed that OPC and SP media resulted in changes in gene expression consistent with a CNS-mimicking microenvironment in contrast to MM.

Interestingly, OPC differentiation medium induced a polarization towards an M2 phenotype, compared to MM, with increased expression of CHI3L1, a chitinase like protein associated with the induction of oligodendrogenesis^29^.

Importantly, factors secreted by MoCs primed in either SP or OPC differentiation media induced a switch from a proliferative (KI67) towards a regenerative (gliogenesis and neurogenesis) program in NSCs. This is particularly interesting as the neurogenic and gliogenic effect as well as the proliferation-inhibiting effects of MoC-conditioned medium were absent when MoCs were cultured in media that does not mimic the NSC microenvironment (MM), indicating the importance of the microenvironment on monocytic secretion of potentially paracrine factors. Within this scope, several studies in recent years looking to investigate the interplay between NSCs or oligodendrocyte precursor cells and infiltrating immune cells have contributed to our current understanding of immune contribution to CNS repair and show the importance of immune cells as central cellular targets to induce or repress CNS regeneration^12,53^. Within a murine model of ischemic stroke, the in vivo depletion of circulating monocytes impaired long-term recovery, and their contribution to repair was accompanied with a switch from a proinflammatory towards an anti-inflammatory phenotype^65^. Contrarily, the depletion of circulating monocytes early after stroke promoted neurogenesis from endogenous NSCs in mice^66^. Kotter et al. found that depletion of circulating monocytes impaired oligodendrocyte precursor cell differentiation and remyelination^67^ in vivo. Furthermore, Möhle et al. identified Ly6C^hi^ monocytes to be crucial players in adult hippocampal neurogenesis^68^. Despite ample in vivo work, current in vitro setups don’t fully characterize all the factors at play; the current assays do not closely resemble the physiology and pathophysiology of the tissue, resulting in the loss of potential insights. We show that both the microenvironment in which monocytes were primed to secrete paracrine factors, as well as the basal medium for NSC culture determine the sensitivity with which MoC-induced alterations in proliferation and differentiation can be detected. Interestingly, using either B27 or NDM allows the detection of MoC-induced changes in proliferation, but both media are distinct in their ability to allow the detection of MoC-induced NSC differentiation—an effect that may be mediated by the distinct composition of vitamins, inorganic acids, and thymidine concentration on NSC-intrinsic properties.

In keeping with this finding, we further show that the susceptibility of NSCs to MoC-induced changes in proliferation and gliogenic vs neurogenic differentiation was altered by the passage number of the respective NSCs.

Consistent with previous findings by Alisch et al.^55^, we also observe a loss of stem cell identity (self-renewal capacity), accompanied with a decrease in pro-neurogenic (DCX) and an increase in pro-oligodendrogenic (NG2) fate choice in high passage NSCs. Contrary to our expectations, low passage NSCs produced significantly more A2B5 + cells compared to mid passage NSCs (data not shown).

While A2B5 positivity includes all (macro) glial-restricted progenitors, NG2 positivity specifically marks oligodendroglia restricted progenitor cells^69^.

Taken together, our results indicate a general increase in proliferation capacity and a decrease in the overall differentiation capacity (overall gliogenic and neurogenic) in mid passage compared to low passage cells, while an oligodendrogenic restricted phenotype may increase proportionally to the passage number. Importantly, exposure of low passage NSCs to MoC-conditioned medium affected proliferation, gliogenic and neurogenic fate, while mid passage NSCs were not susceptible to MoC-induced gliogenesis.

It is important to emphasize that all effects observed by the conditioned-media are related to the composition of the different media. First and foremost, we have shown how media itself, usually taken for granted, can greatly affect the results of an assay. Thus, our data provides a starting point for follow-up studies investigating monocyte-derived factors in inflammatory or anti-inflammatory conditions, and/or using cells directly derived from patients such as neural stem cells generated from patient-derived iPSC.

Lastly, we carried out all experiments in a high throughput set up, optimizing the assay for low variability and utilizing statistical methods that are thorough and robust. The system is ready for the use of material from large patient cohorts aiming to investigate the interplay between immune cells and NSCs. In this line, the use of cerebrospinal fluid, or medium conditioned with immune-cells of a MS patient could shed light on how NSCs are affected by disease. Moreover, comparing CSF or cells from different disease stages might elucidate mechanisms of regeneration failure seen in chronic forms of MS. Ultimately this assay could be expanded to the use of patient iPSC-derived NSCs, creating a fully personalized model that could assess the efficacy of tailored treatments in promoting regeneration. The high throughput set-up also optimally positions this assay to be used in drug discovery.

While no simple immunofluorescence panel will allow for a thorough characterization of all cells present in the culture, a simple panel with few markers can still provide valuable insight into the general effects of immune-secreted factors on NSC proliferation and differentiation. Alternatively, more advanced high-plex imaging techniques, or single cell sequencing are needed for a full characterization of the cells.

It is also important to note that while the short differentiation time allows for rapid screening it does not allow for terminal differentiation of the progeny. However, such a screening tool can potentially identify factors that can further be investigated in long-term differentiations to assess for instance whether terminal differentiation is affected, what types of neurons are generated, or the myelinating capacity of the oligodendrocytes generated.

As a last caveat, it is important to remark the incomplete information on the composition of many of the substances and media used. We are therefore unable to draw conclusions about what specific factors of the media may contribute to the effects reported in this study. Nevertheless, given the widespread use of these products, these effects must be considered when developing differentiation assays.

In conclusion, by integrating both cell intrinsic and CNS-specific microenvironmental parameters, we developed a robust and fully human assay platform that is optimized for mimicking the physiological changes caused by monocytes and MoCs entering the CNS. Together, we provide specific building blocks to tailor an assay for human immune cell-induced CNS regeneration in a dish to best fit specific areas of interest, such as proliferation, neurogenesis or gliogenesis.

### Supplementary Information

Supplementary Figure 1.

Supplementary Figure 2.

## Data Availability

The datasets used and/or analyzed during the current study are available from the corresponding author upon reasonable request.

## Abbreviations

APC: Antigen-presenting cell
BSA: Bovine serum albumin
CCL: C–C motif chemokine ligand
CD: Cluster of differentiation
CHI3L1/2: Chitinase-3-like protein 1/2
CHID: Chitinase Domain Containing 1
CNS: Central nervous system
CSF1R: Colony stimulating factor 1 receptor
DAPI: 4′,6-Diamidino-2-phenylindole
DCX: Doublecortin
DMSO: Dimethyl sulfoxide
EGF: Epidermal growth factor
FCS: Fetal calf serum
FGF: Fibroblast growth factor
LMM: Linear mixed models
MACS: Magnetic cell separation
MM: Monocyte medium
MoC: Monocyte-derived cell
N2: Nitrogen
NDM: Neural differentiation medium
NI: Neuroinflammation
NG2: Neural/glial antigen 2
NSC: Neural stem cell
P: Passage
PBS: Phosphate-buffered saline
PBMC: Peripheral blood nuclear cell
PFA: Paraformaldehyde
SD: Standard deviation
STAT: Signal transducer and activator of transcription
SP: StemPro differentiation medium
