# Supplementary figures and images for: Establishment of a high-content compatible platform to assess effects of monocyte-derived factors on neural stem cell proliferation and differentiation
Source: Sci Rep. 2024 May 28;14:12167. doi: 10.1038/s41598-024-57066-2 (PMC11133477; doi:10.1038/s41598-024-57066-2)

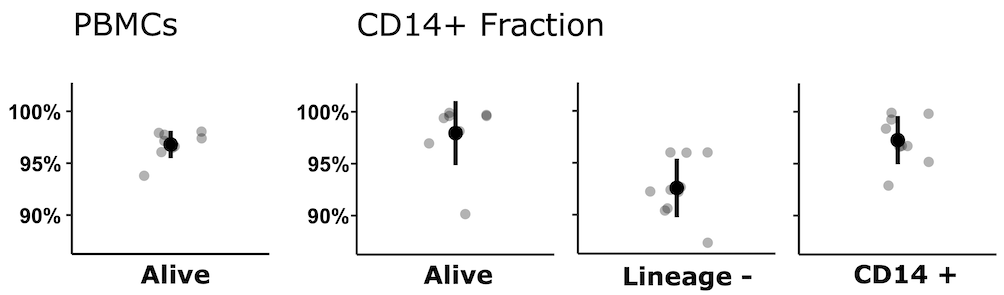

Supplement: Supplementary file 1 — Supplementary Figure 1. [file 41598_2024_57066_MOESM1_ESM.tiff]

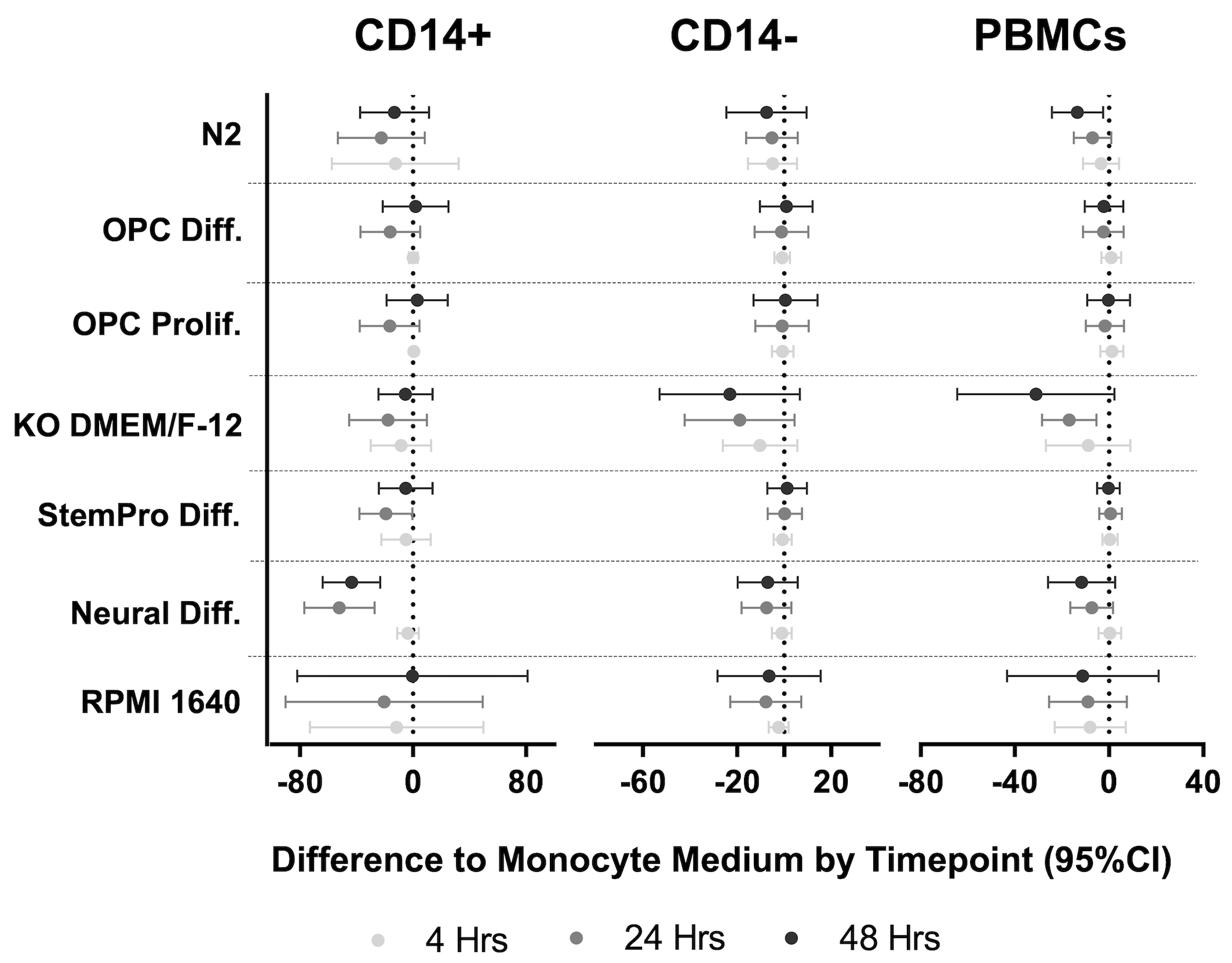

Supplement: Supplementary file 2 — Supplementary Figure 2. [file 41598_2024_57066_MOESM2_ESM.tiff]
